# Direct Rapid Identification from Positive Blood Cultures by MALDI-TOF MS: Specific Focus on Turnaround Times

**DOI:** 10.1128/spectrum.01103-21

**Published:** 2021-12-15

**Authors:** Hazan Zengin Canalp, Banu Bayraktar

**Affiliations:** a Sisli Etfal Training and Research Hospital Department of Medical Microbiology, University of Health Sciences, Istanbul, Turkey; Houston Methodist Hospital

**Keywords:** blood culture, MALDI-TOF MS, bacteria, rapid identification, turnaround time

## Abstract

Early availability of pathogen identification in bloodstream infections has critical importance in patients' management. This study investigated the accuracy and feasibility of the direct rapid identification (RID) method from positive blood cultures (BCs) by MALDI-TOF MS and its impact on the turnaround time (TAT) compared to the short-term incubation routine identification (SIRID) method. Pellets prepared from 328 BCs using a serum separator tube in the RID method and colonies on agar plates in the SIRID method were identified with MALDI Biotyper. BCs on weekdays from 6 a.m. to 4 p.m. were defined as the daytime signal group (DSG); BCs from 4 p.m. to 6 a.m. were defined as the night signal group (NSG). Comparison between the two methods was performed with 310 monomicrobial BCs. Two hundred ninety-five (95.2%) monomicrobial BCs yielded an identification result with the RID method. Of the 295 BCs, 289 (97.9%) were identified correctly at the species level, 4 (1.4%) were at the genus level, and 2 (0.7%) were misidentified. In the RID method, at score cutoff values of 1.2, 1.3, 1.4 and 1.5, the rates of correct identifications at the species level were 97.9%, 98.9%, 99.3%, and 100%, respectively. The mean TAT in the DSG was significantly lower (*P* < 0.001) in the RID method (mean: 2.86 h; 95% CI: 2.65 to 3.07) compared to the SIRID method (mean: 19.49 h; 95% CI: 18.08 to 20.89). Correct identification rates at the species level were 100% in Gram-negative bacteria, 88.9% in Gram-positive bacteria, and 93.2% of all BCs isolates with the RID method. The TAT was improved remarkably in DSG, which might contribute to empirical antibiotic therapies of patients.

**IMPORTANCE** Using MALDI-TOF MS directly from BCs reduces the time required for pathogen identification, and the TATs for final identification have been compared with overnight incubation from solid media in previous studies. However, identification from a short incubation of agar plates has been increasingly accepted and successfully implemented in routine laboratories, but there is no data comparing direct MALDI-TOF MS with the short-term incubated agar plates. Our study showed that the TAT improved remarkably by applying a RID method by MALDI-TOF MS twice a day periodically when compared to the SIRID method.

## INTRODUCTION

Bloodstream infections cause serious mortality and morbidity, although mortality risk and length of hospital stay could decrease by rapid identification of pathogens in blood culture (1). Blood culture is the gold standard in the diagnosis of bloodstream infections. The matrix-assisted laser desorption ionization-time of flight mass spectrometry (MALDI-TOF MS) system is used in the identification of pathogens in clinical microbiology laboratories. In the standard practice of blood culture, positive blood culture bottles are subcultured on solid media and, following overnight incubation, growing colonies are used for identification by the MALDI-TOF MS system.

The rapid identification of a bacterial pathogen in blood culture is critical in supporting an effective antimicrobial stewardship program and patient management. By shortening the incubation time of subculture agar plates for routine identification, bacteria can be identified earlier with MALDI-TOF MS (2–4). After positive signaling in blood culture, it is aimed to identify the pathogen within the same day. Although there is a consensus that molecular tests would be the best method for pathogen identification in blood culture, their widespread use varies from laboratory to laboratory in different countries. The most important factor causing this is the high cost of the test. Another factor is the identification of only a limited number of species included in those panels (5–9).

Microorganisms can be identified directly from a positive blood culture bottle by the MALDI-TOF MS system using “in-house” methods or with commercial kits and additional software modules, such as the MBT-Sepsityper (Bruker Daltonics). While system software changes, such as the MBT-Sepsityper module, can be used for reading purposes, many laboratories have adopted in-house, validated read methods by using standard software-based rules for acceptance criteria that do not require switching software (9–11).

In previous studies investigating the rapid identification of bacteria directly from blood culture bottles by MALDI-TOF MS, the turnaround times (TAT) for final identification were compared with overnight incubation from solid media. However, there is no data comparing the results with short-term incubated agar plates (12–15). This study is aimed to demonstrate the accuracy and feasibility of rapid direct identification from positive blood cultures by MALDI-TOF MS and its impact on the TAT compared to the short-term incubation method.

## RESULTS

The 328 blood cultures gave a positive signal from 231 aerobic and 97 anaerobic blood culture bottles. The daytime signal group (DSG) consisted of 152 blood culture bottles, and the night signal group (NSG) consisted of 176 bottles.

### SIRID results.

A total of 328 blood cultures consisting of 120 Gram-negative, 190 Gram-positive monomicrobial, 16 polymicrobial, and two yeast were correctly identified at the species level by the short-term incubation routine identification (SIRID) method. The most common bacterial isolates were coagulase-negative Staphylococci (*n* = 106), Enterobacterales (*n* = 98), and S. aureus (*n* = 36).

### Gram-negative SIRID results.

A total of 120 Gram-negative monomicrobial blood culture isolates were identified at the species level by the SIRID method. The most common strains were Escherichia coli (*n* = 61), Klebsiella pneumoniae (*n* = 28), and Acinetobacter baumannii (*n* = 10). MALDI Biotyper scores were between 2.00 and 2.53 in the SIRID method with a mean value of 2.29.

### Gram-positive SIRID results.

A total of 190 Gram-positive monomicrobial blood culture isolates were identified at the species level by the SIRID method. The most common strains were Staphylococcus epidermidis (*n* = 43), Staphylococcus hominis (*n* = 38), and Staphylococcus aureus (*n* = 36). MALDI Biotyper scores of Gram-positive isolates were between 2.00 and 2.404 in the SIRID method with a mean value of 2.108.

### RID method results.

**FIG 1:**
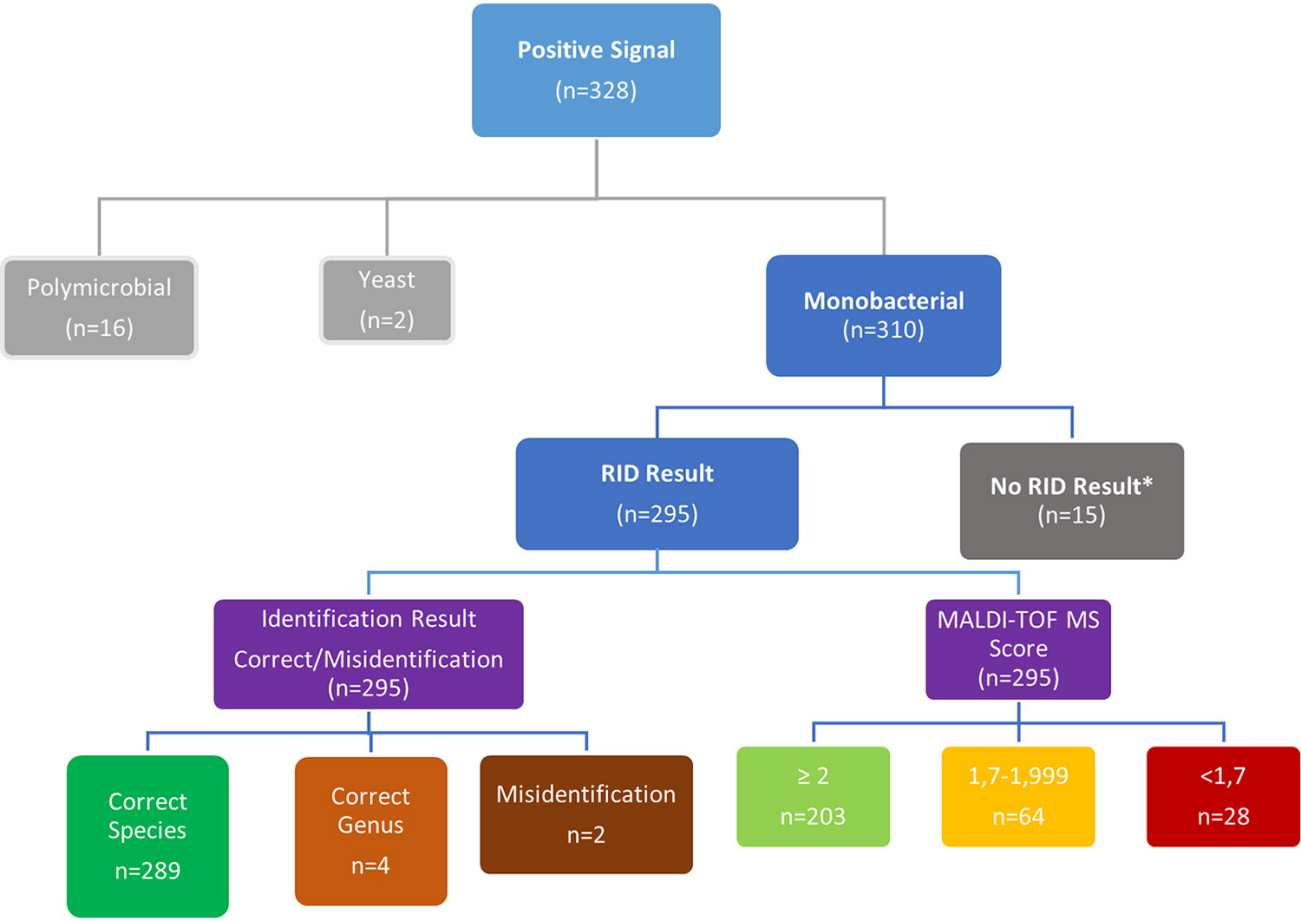
Rapid identification (RID) method results of positive signaling blood cultures. *, No peak in MALDI-TOF MS analysis of the RID method.

Of the 310 monomicrobial samples, 295 (95.2%) yielded a result with the direct rapid identification (RID) method, and 15 (4.8%) did not yield any result. The RID method results of positive signaling blood cultures are summarized in Figure 1.

The rate of correct identification at the species level in monomicrobial samples was 93.2% (289/310). Among the correctly identified isolates (*n* = 289), the rates of identification scores greater than 2.0, between 1.7 and 1.999, and less than 1.7 were 70.2%, 22.2%, and 7.6%, respectively. Out of 28 samples with a rapid identification score of less than 1.7, 22 were correctly identified at the species level, 4 were correct at the genus level, and 2 samples were misidentified. The RID method scores of four isolates were correct at the genus level, and two misidentified isolates were less than 1.5. For different scores taken as threshold values in the RID method, the rates of correct identification at the genus and species levels are in Table 1. The correct identification rate with the rapid method was 100% at the species level in isolates with a score of 1.5 and above.

**TABLE 1:** Correct identification rates according to score threshold values in RID

RID result^*a*^	MALDI-TOF MS score threshold value								
	≥ 1.2	≥ 1.3	≥ 1.4	≥ 1.5	≥ 1.6	≥ 1.7	≥ 1.8	≥ 1.9	≥ 2.0
Correct at species level	97.97%	98.96%	99.3%	100%	100%	100%	100%	100%	100%
Correct at genus level	99.32%	99.65%	100%	100%	100%	100%	100%	100%	100%

a RID, rapid identification.

### Gram-negative RID results.

The rate of correct identification at the species level in Gram-negative isolates was 100% with the RID method. There were no misidentified or unidentified isolates. The distribution of the results by species from the RID method is in Table 2. The mean value of the MALDI Biotyper score from the RID method was 2.202 (95% CI: 2.174 to 2.230). Stenotrophomonas maltophilia had the lowest score with 1.702 and K. pneumoniae had the highest score with 2.480.

**TABLE 2:** Number of isolates from the rapid identification (RID) results of Gram-negative bacteria

Species	Correct at species level			Correct at genus level	Misidentified	Unidentified	Total (%)
	MALDI-TOF MS score						
	< 1.7	1.7-1.99	≥ 2				
E. coli		1	60				61 (50.8)
K. pneumoniae		1	27				28 (23.3)
A. baumannii		4	6				10 (8.3)
P. aeruginosa			5				5 (4.2)
Burkholderia cepacia			2				2 (1.7)
E. cloacae			2				2 (1.7)
K. variicola			2				2 (1.7)
Salmonella *spp.*			2				2 (1.7)
Aeromonas hydrophilia			1				1 (0.8)
Bacteroides fragilis			1				1 (0.8)
K. aerogenes		1	1				2 (1.7)
P. mirabilis			1				1 (0.8)
S. maltophilia		1	1				2 (1.7)
Pseudomonas *sp.*		1					1 (0.8)
Total (%)		9 (7.5)	111 (92.5)				120

### Gram-positive RID results.

The rate of correct identification of the RID method in Gram-positive isolates was 88.9% (169/190) at the species level, was 2.1% (4/190) at the genus level, while 1.1% (2/190) were misidentified, and 7.9% (15/190) were unidentified. The distribution of the results of the RID method by species is in Table 3.

**TABLE 3:** Number of isolates from the rapid identification (RID) results of Gram-positive bacteria

Species	Correct at species level			Correct at genus level			Misidentified			Unidentified	Total (%)
	MALDI-TOF MS score			MALDI-TOF MS score			MALDI-TOF MS score				
	< 1.7	1.7-1.99	≥ 2	< 1.7	1.7-1.99	≥ 2	< 1.7	1.7-1.99	≥ 2		
S. epidermidis	9	20	8	1						5	43 (22.6)
S. hominis	1	12	20	2						3	38 (20)
S. aureus	2	11	19				1			3	36 (18.9)
E. faecalis	1		16								17 (8.9)
E. faecium			5								5 (2.6)
S. haemolyticus	2	4	4	1						2	13 (6.8)
S. pneumoniae			8								8 (4.2)
S. capitis	3	1	2							1	7 (3.7)
Corynebacterium striatum		2	2								4 (2.1)
Corynebacterium afermentans			1								1 (0.5)
Corynebacterium jeikeum										1	1 (0.5)
S. gallolyticus	1	1									2 (1.1)
S. pyogenes		1	1								2 (1.1)
Brevibacterium ravenspurgense		1									1 (0.5)
Clostridium ramosum			1								1 (0.5)
Clostridium tertium			1								1 (0.5)
Granulicatella adiacens			1								1 (0.5)
Pseudoglutamicibacter cumminsii		1									1 (0.5)
S. agalactiae			1								1 (0.5)
S. anginosus	1										1 (0.5)
S. auricularis		1									1 (0.5)
S. cohnii	1										1 (0.5)
S. mitis			1								1 (0.5)
S. pettenkoferi							1				1 (0.5)
S. simulans			1								1 (0.5)
S. warneri	1										1 (0.5)
Total	22	55	92	4			2			15	190
(%)	(11.6)	(28.9)	(48.4)	(2.1)			(1.1)			(7.9)	

The mean value of the MALDI Biotyper scores in the RID method was 1.940 (95% CI: 1.899 to 1.981). Enterococcus faecalis had the highest score with 2.478, and S. epidermidis had the lowest score with 1.258. All four isolates that could be identified correctly at the genus level were coagulase-negative *Staphylococci*. The correct identifications of the two isolates misidentified by the RID method were S. aureus and S. pettenkoferi. Both isolates were misidentified as Mannheimia haemolytica by the RID method (scores of 1.251 and 1.389, respectively).

### Polymicrobial cultures.

The RID method had an accurate identification for one of the mixed bacterial isolates from 14 out of 16 polymicrobial cultures. The RID method results of the polymicrobial blood cultures are in Table 4.

**TABLE 4:** Rapid identification results of polymicrobial blood cultures

Rapid identification result	Correctly identified species MALDI-TOF MS Score			Misidentified	Unidentified additional isolates
	<1.7	1.7-1.99	≥ 2		
S. epidermidis		1			S. hominis
S. hominis		1			S. epidermidis
S. epidermidis		1			Corynebacterium amycolatum
S. hominis			1		Corynebacterium minutissimum
S. aureus	1				S. hominis
S. haemolyticus			1		S. epidermidis
S. epidermidis	1				S. haemolyticus
E. faecalis			1		S. epidermidis, S. haemolyticus
S. haemolyticus			1		S. capitis
S. warneri		1			S. capitis
S. haemolyticus			1		P. aeruginosa
Rhizobium radiobacter				1*	S. sanguinis, S. haemolyticus
E. coli			1		S. epidermidis, Bacillus megaterium
P. aeruginosa			1		A. baumannii
A. baumannii		1			K. pneumoniae
Unidentified					S. aureus, S. haemolyticus

*MALDI Biotyper score 1.42.

### TAT.

The mean TAT of the RID method was significantly shorter than the SIRID method (*P* < 0.001). Table 5 shows the comparison of the mean TAT in hours for both methods and signal groups. In the DSG, the mean TAT of the RID method was 16.63 h lower than the SIRID method (*P* < 0.001). There was no significant difference between the mean TAT values of the identification methods for the NSG (*P* = 0.146).

**TABLE 5:** Mean TATs in hours according to the signal group in RID and SIRID^*a*^

Signal group	TAT of RID Mean±SD (95% CI)	TAT of SIRID Mean±SD (95% CI)
DSG (*n* = 132)	2.86 ± 1.22 (2.65, 3.07)	19.49 ± 8.18 (18.08, 20.89)
NSG (*n* = 163)	11.96 ± 4.14 (11.32, 12.6)	14.36 ± 7.06 (13.27, 15.45)
Total (*n* = 295)	7.88 ± 5.54 (7.25, 8.52)	16.66 ± 7.99 (15.74, 17.57)

a DSG, daytime signal group; NSG, night signal group; TAT, turnaround time; RID, rapid identification; SIRID, short-term incubation routine identification.

**FIG 2:**
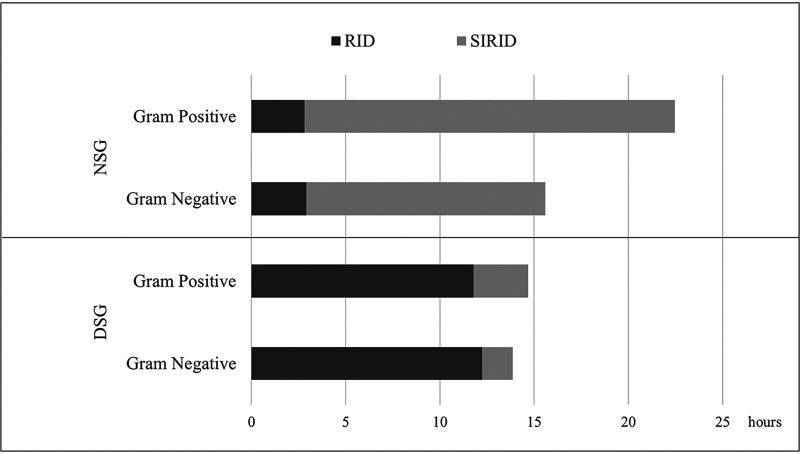
Distribution of mean TAT of RID and SIRID methods in DSG and NSG according to Gram staining. TAT, turnaround time; RID, rapid identification; SIRID, short-term incubation routine identification; DSG, daytime signal group; NSG, night signal group.

Figure 2 shows the mean TAT of both methods in both signal groups according to Gram staining. In the DSG, the mean TAT of the RID method was 12.67 h shorter in Gram-negative bacteria and 19.65 h in Gram-positive isolates when compared to the SIRID method. In the NSG, the mean TAT of the RID method was 2.9 h less in Gram-positive isolates and 1.62 h in Gram-negative isolates when compared to the SIRID method.

## DISCUSSION

The RID method performed using MALDI-TOF MS yielded 93.2% correct identification at the species level in 295 monomicrobial growth out of 328 positive blood culture bottles regardless of the score. Our study differs from previous studies in the direct identification of blood cultures by MALDI-TOF MS because we focused on the TAT of the RID and SIRID methods. The mean TAT was shortened by 16.63 h by the RID method compared to that of the SIRID method. Although the SIRID method improved the mean TAT (16.66 h for the SIRID method) compared to the MALDI-TOF identification after an overnight incubation from solid media, it still lagged the targeted TAT (1 to 8 h [16]) in blood culture within the same day to contribute to patient management.

The separation of blood cell components from the positive blood culture medium is the troublesome and critical part of the direct MALDI-TOF MS identification. Various chemical buffers, such as saponin, EDTA, NaHCO_3_, NH_4_Cl, KHCO_3_, Triton X-100, and Tween 80, or serum separator tubes have been employed for different sample preparation and extraction procedures to get rid of the blood cells (17–26). The rates of correct identification at the species level change between 60% and 99%, and hands-on times vary between 15 and 40 min for various methods (9).

The methods with serum separator tubes overcome the extended TAT problem due to preparation methods that encompass repetitive washing and centrifugation steps with buffers. Additional extraction steps, such as acetonitrile and formic acid, for improving identification scores in MALDI-TOF MS are time-consuming. Therefore, for practicality and shortening the hands-on time in our study, serum separator tubes and the direct formic acid extraction method on the target plate were preferred for the RID method sample preparation.

In other studies, using different lysis methods (Saponin, NH_4_Cl, KHCO_3_, and Triton X-100), the rates of species identification varied from 90% to 94% in Gram-negative and 73% to 85% in Gram-positive bacteria and were lower than our study (19, 23, 27–29). Concordant with our data, two studies reported rates of 100% correct identification at the species level in Gram-negative bacteria with 5% sodium dodecyl sulfate (25) and hemolysis buffer containing NH_4_Cl, NaHCO_3_, and EDTA (20). In previous studies using serum separator tubes, rates of correct identification of Gram-negative bacteria varies from 93% to 100% and identification of Gram-positive varies from 83% to 92% (17, 21, 30). Our identification rates with serum separator tubes were 100% in Gram-negative and 88.9% in Gram-positive, which were similar to Barnini et al. (17).

Several studies showed that the identification at the species level was reliable if the MALDI Biotyper score was 1.5 or above (13, 23). Another study reported that a score threshold of 1.4 might be valid at the species level (17). Consistent with these studies, we obtained 100% accurate identification at the species level with a threshold score of 1.5 or above and 100% correct identification at the genus level with threshold scores of 1.4.

A software module recently developed for the MALDI Biotyper, the MBT-Sepsityper module, score threshold values were lowered and determined reliably at 1.6 at the genus level and 1.8 at the species level, which was previously accepted at 1.7 and 2.0, respectively, for identification from the colony in the standard module (31, 32). For direct identification from blood culture bottles, the MBT-Sepsityper module provided significantly higher accuracy of the identification rates compared to the standard software module (11, 33).

We could not identify 4.8% (15/310) of the monomicrobial cultures with the RID method. These isolates were Gram-positive bacteria, consisting of 11 coagulase-negative Staphylococci, 3 S. aureus, and 1 Corynebacterium jeikeum. The isolates unidentified by the RID method were predominantly coagulase-negative Staphylococci, which was similar to other studies (17). The rate of unidentified isolates has been reported to be between 10 and 13% in studies without an extraction protocol (21, 30). In a study, where only an extraction protocol was used in Gram-positive bacteria, the rate of unidentified isolates was reported to be 6% (17). In our study, the rate of unidentified isolates was 4.8% with an extraction protocol for both Gram-negative and Gram-positive bacteria and was lower than in these studies.

The misidentification rate varies between 0 and 4% in the literature (17, 21, 30). The misidentification rate was low in our study (0.6%). The identification of two isolates that were misidentified as Mannheimia haemolytica with the RID method were Staphylococcus aureus and Staphylococcus pettenkoferi. Mannheimia haemolytica is a Gram-negative bacterium and is not known as a human pathogen. If the identification score was low and the species' name is not an expected human pathogen, the RID method results should be interpreted cautiously together with Gram-stain to prevent possible reporting of misidentification. Most of the unidentified isolates by the RID method in the polymicrobial cultures were Gram-positive bacteria, and these isolates were commonly coagulase-negative Staphylococci alike to other studies (17, 21).

Although some of the polymicrobial cultures could be detected by Gram staining characteristics, the result was determined by culture growth in solid media. When processing bacteria in the RID method, it should be taken into consideration that the polymicrobial culture may be incorrectly misinterpreted as monomicrobial if the polymicrobial culture consists of isolates with similar Gram staining characteristics and morphology.

Due to the low number of isolates, yeasts were excluded from the evaluation of the RID method. During the study period, only two yeasts, Candida parapsilosis and Rhodotorula mucilagunosa, grew from blood cultures. These isolates were identified as Candida parapsilosis (score of 1.2) and Candida famata (score of 1.3) with the RID method. Both scores were low, and the latter identification was incorrect.

Several studies showed that the direct identification from the blood culture bottle shortened the TAT by 1 to 29 h compared with MALDI-TOF identification after an overnight incubation from solid media (12, 14, 15). Other studies reported that the direct identification from blood culture by MALDI-TOF MS saves 6 to 48 h compared to biochemical identification methods (13, 34).

The mean TAT of 16.66 h in the SIRID method was shorter compared to previous studies using identification methods after overnight incubation from solid media (12, 14, 15). The reason for this was that subcultures in solid media were evaluated twice a day at 4 to 5 h intervals in the SIRID method and if visible growth was present, proceeded to pathogen identification. We further reduced the mean TAT from 7.9 h with the RID method to 2.9 h in DSG. While applying the rapid identification method in our study, we preferred the twice a day batch testing that we could integrate into the routine workflow of our laboratory. When the samples were examined in two separate groups as the DSG and NSG, the TATs of the RID method were significantly shorter in the DSG but not in the NSG. Because the RID method was performed twice daily during working hours, the bottles in the NSG were included in the RID and SIRID methods the next day at almost the same time. The study showed that the RID method has a shorter mean TAT than the SIRID method, and significant efficiency was achievable even by performing the RID method twice a day during working hours. Under optimal conditions, prompt identification of each vial with a positive signal will result in an even greater improvement in TAT.

The decrease of the TAT was prominent in Gram-negative bacteria compared to Gram-positive bacteria due to the difference in growth rates on agar plates between the two groups. Gram-negative bacteria grow faster than waiting for the results of the SIRID method on the same day. On the other hand, the SIRID method is delayed to the next day for Gram-positive bacteria. In the NSG, the agar plates that were subcultured at night showed sufficient growth in the morning regardless of the Gram characteristics of bacteria. Therefore, all bacterial colonies were processed at almost the same time with both the RID and SIRID methods. Thus, TAT for the Gram-negative and Gram-positive bacteria were close to each other in both methods.

Three anaerobic bacteria, Clostridium ramosum, Clostridium tertium, and Bacteroides fragilis, were isolated during the study period. These isolates were identified with scores above 2.0 and at 22 to 53 h earlier than the routine method with the RID method. Thus, the RID method also saves significant time in terms of reporting for anaerobic infections.

The limitation of our study is that it focused only on improvements in TAT and the accuracy of the identification, but the contribution of the RID method to the clinical outcome was not investigated.

In conclusion, by processing this easy-to-apply, twice-a-day, periodic RID method from positive signaling blood culture bottles, TAT was improved remarkably without requiring any additional equipment and might contribute positively to patient management.

## MATERIALS AND METHODS

### Study design.

This study was approved by the ethics committee of Sisli Hamidiye Etfal Training and Research Hospital (record number 2631). In the medical microbiology laboratory of a training and research hospital, 328 blood culture bottles (BD Biosciences Bactec Plus Aerobic/F and BD Bactec Plus Anaerobic/F) that signaled positive by using Bactec FX blood culturing instrument between October 2019 to July 2020 were included in the study. The first positive signaled bottle out of the simultaneously collected blood culture sets was considered for each patient. Furthermore, positive blood cultures were divided into two groups according to their signaling time during the incubation period. Positive signaling blood culture bottles on weekdays between 6 a.m. to 4 p.m. were defined as the DSG and positive signaling bottles from 4 p.m. to 6 a.m. were defined as the NSG.

### Short-term incubation routine identification method.

After positive signaling of blood culture bottles, Gram staining, and subcultures on blood agar and chocolate agar (additional Schaedler agar if the bottle was anaerobic) were performed routinely. Blood agar was incubated at atmospheric conditions, chocolate agar was incubated at a 5% CO_2_ environment, and Schaedler agar was incubated at anaerobic conditions at 35 to 37°C.

The incubated plates were routinely checked for colony growth twice daily at 9 a.m. and 2 p.m.. The SIRID method using MALDI-TOF MS identified visible microbial growth on the agar plates. The microbial growth on agar plates was taken with a wooden stick and spotted onto stainless steel target plate three times. One microliter of 70% formic acid (catalog no. 27001, Sigma-Aldrich, Germany) was added for extraction and left to dry at room temperature, followed by one microliter of matrix solution (α-Cyano-4-hydroxycinnamic acid [HCCA], Bruker Daltonics, Germany) was added and allowed to dry. Afterward, the target plate was placed in MALDI Biotyper (Bruker Biotyper, Bruker Daltonics, Germany) and analyzed.

### Rapid identification method.

**FIG 3:**
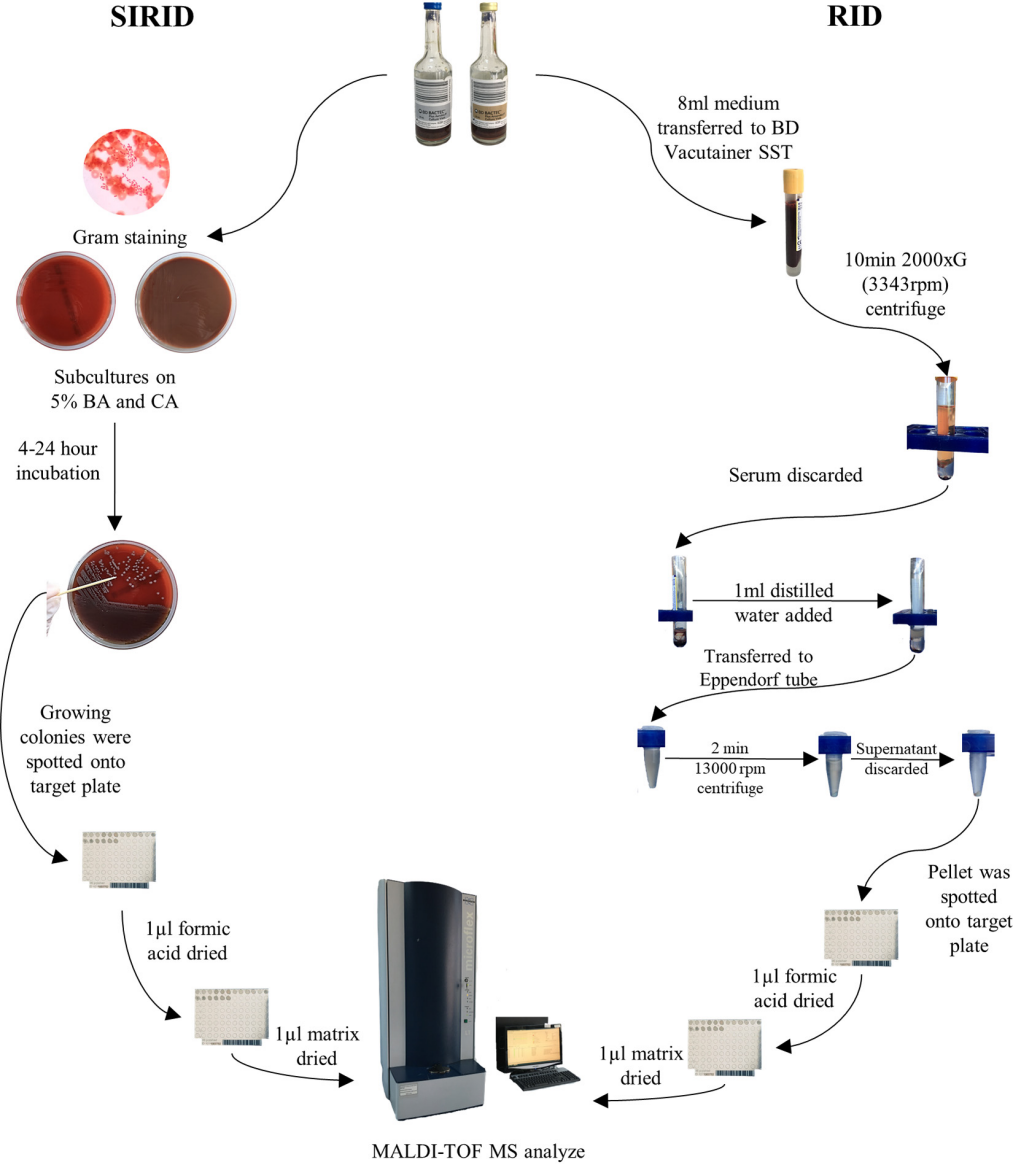
Flow chart of short-term incubation routine identification (SIRID) and rapid identification (RID) methods. SST, serum seperator tube; BA, Blood agar; CA, Chocolate agar.

The positive signaling blood culture bottle proceeded the RID after the SIRID procedures. The RID method procedures were carried out twice daily during working hours (11 a.m. and 4 p.m., and positive signals after 4 p.m. were processed at 11 a.m. in the next morning). The bottles stayed at room temperature until they proceeded to the RID method. A modified version of the method, previously defined by Barnini et al. (17) was used for the RID method. An 8 mL medium from a positive signaling blood culture bottle was taken to the serum separator tube (BD Vacutainer SSTII Advance), followed by centrifugation, and the extraction steps are shown in Figure 3. Prepared pellets were analyzed on the MALDI-TOF MS.

### MALDI-TOF MS system.

All identification procedures were performed by MALDI Biotyper (Bruker Biotyper; Bruker Daltonics, Bremen, Germany) using a stainless-steel target plate. Spectra were analyzed in Bruker Biotyper 3.1 BDAL (RUO) version 8.0 (DB-7854 MSP) library with FlexAnalysis 3.4 software. Following the manufacturer’s recommendations, a score of 2.0 or above indicated correct identification at the species level, scores between 1.700 and 1.999 indicated identification at the genus level, and a score below 1.7 was considered unreliable.

### Data analysis.

For each isolate, all scores obtained in the SIRID and RID methods were, and the highest scores among the same genus and species level were selected to compare the two methods. The results with a score of 2.0 or above using the SIRID method were accepted as the reference identification and the accordance of the RID method with the reference evaluated. The RID method results were categorized as correct at the species level, correct at the genus level, or a misidentified result according to concordant with the reference.

The positive signal, RID method, and SIRID method date and time were recorded in excel sheets. The TATs for each method were calculated in units of hours by subtracting the positive signaling time from the RID and SIRID method results time. Only monomicrobial samples were included in statistical analysis, polymicrobial samples were excluded. IBM SPSS Statistics 26.0 for Windows program was used for statistical analysis.

Descriptive statistics were given as numbers and percentages for categorical variables, mean, standard deviation, minimum, and maximum for numerical variables. Continuous variables were compared with paired sample *t* test when there was a normal distribution, and the Wilcoxon test when there was an asymmetric distribution. All analyses were performed to obtain a 95% confidence interval. The statistical significance level of alpha was accepted as *P* < 0.05.
